# Comparative Analysis of Learning Curves in Robotic Versus Laparoscopic Cholecystectomy: A Systematic Review

**DOI:** 10.7759/cureus.67468

**Published:** 2024-08-22

**Authors:** Md Rezaul Karim, Amos E Kong, Noor Mohammad, Riddhi N Shah, Bijendra Patel

**Affiliations:** 1 Surgery, Barts Cancer Institute, Queen Mary University of London, London, GBR; 2 Surgical Science, Barts Cancer Institute, Queen Mary University of London, London, GBR; 3 Trauma and Orthopaedics, Royal London Hospital, London, GBR; 4 Surgery, Royal London Hospital, London, GBR; 5 Surgery, Queen Mary University of London, London, GBR

**Keywords:** lap cholecystectomy, robotic cholecystectomy, robotic assisted cholecystectomy, robotic surgery, laparoscopic surgery, learning outcome, learning curve

## Abstract

Robotic surgery has undergone much development and increased use over the years; it has offered many benefits for the operating surgeon compared to the more restrictive nature of conventional laparoscopic surgery (CLS) which is the current standard of care. However, to the best of our knowledge, no studies have attempted to draw a comparison between the two in terms of the cases required for the learning curve to be achieved.

The systematic review was performed at Barts Cancer Institute. A search of Cochrane, PubMed and Embase was made on 15 March 2024. Screening and risk of bias were done by two reviewers. Screening was done via the eligibility criteria by two reviewers. Data collection was done using Excel (Microsoft® Corp., Redmond, USA) and information was double-checked by another reviewer and transferred into a tabulated format.

Seventeen studies were included, with the learning curve reported in 14 studies. The cases required to achieve the learning curve for multiport robotic cholecystectomy (MRC) ranged from 16 to 134 and for single-site robotic cholecystectomy (SSRC), it ranged from 10 to over 102 cases. Conventional laparoscopic cholecystectomy (CLC) was from 7 to 200. The improvement in operating times was measured in very different ways and was reported in 10 of the 17 studies.

The studies that were available had a high level of heterogeneity making it difficult for comparisons to be made between studies. Several studies included only one surgeon resulting in the sample size of surgeons being too small and vulnerable to bias. As robotic surgery is still relatively novel, higher-quality studies have to be made in order for more conclusive conclusions to be made on the benefits of the learning curve of MRC and SSRC.

## Introduction and background

Robotic surgery is at the forefront of current surgical innovation and has widened its application over the last decade [1]. It presents many advantages compared to its laparoscopic predecessor. These include its improved degrees of freedom via its endo-wrist design for multiport robots (and newer single port robots), the more comfortable seating position at the console and the 3D view when operating [2]. However, due to its novelty, the learning curve of robotic surgery still hasn’t been fully explored; certain procedures such as distal gastrectomy have shown that the robotic surgical learning curve may possibly be shorter when compared to the laparoscopic option [3]. Laparoscopic cholecystectomy is the gold standard for the treatment of many conditions [4] and has been shown to still hold up compared to its robotic counterpart which tends to be uncommon considering the superiority of current-day robotic surgery [5]. However, as robotic surgery becomes more widely accessible, it will be important to study how the learning curve for robotic surgery for this procedure compares to laparoscopic surgery and whether it follows the same trend as other procedures.

This systematic review aims to compare the learning curve of robotic and laparoscopic cholecystectomy. The population chosen will not be too strict due to the scarcity of papers, thus, studies involving residents and consultants with decades of experience in robotic and laparoscopic cholecystectomy will be included. The learning curve for laparoscopic cholecystectomy will make a good comparator for robotics considering it currently still is the standard of care for this operation [5]. The learning curve can be measured by the number of cases required till there are non-significant changes in the total operating time. Improvement in operating times may not be an ideal way of gauging the improvement; however, it is the most commonly used variable to measure the improvement and ease of an operation which could be linked to the learning curve. Thus, it may answer the question as to whether robotic cholecystectomy will have a faster learning curve as shown in other procedures [3].

## Review

Methods

As per the Preferred Reporting Items for Systematic Reviews and Meta-Analyses (PRISMA) guidelines, we conducted a systematic electronic search from February 2024 to March 2024 to find ongoing and recently completed studies. We used a predefined search strategy to search the Cochrane Library, PUBMED, and EMBASE for the systematic review.

Eligibility Criteria

Due to the scarcity of papers, the exclusion criteria were not stringent. Animal studies, pediatric cases, simulations and single-incision laparoscopic surgery were all excluded from the study. Single-incision laparoscopic cholecystectomy is not the standard of care, so it was decided to exclude it.

Both multiport and single-site robotic cholecystectomy (SSRC) were included as although many older models of single port robotic systems do not have the endo-wrist function, they still share many of the advantages multiport robotic cholecystectomy (MRC) has over conventional laparoscopic cholecystectomy (CLC). Studies directly comparing both robotic and laparoscopic cholecystectomy learning curves were not found; thus, all studies that included the intervention or comparator alone were used for the review. Only studies that measured the learning curve based on the improvement in total operating time and number of cases were included. Only studies published in English literature are included.

Inclusion and exclusion criteria: The inclusion criteria consist of adult patients undergoing cholecystectomy, focusing on MRC and SSRC as interventions, with CLC serving as the control or comparator. The outcomes of interest are papers measuring the number of cases until the total operating time plateaus. The studies included are English papers published from 1997 to 2024, encompassing both randomized controlled trials (RCTs) (though none were found) and non-RCTs. On the other hand, the exclusion criteria eliminate simulations and pediatric cases from the population, single incision laparoscopic cholecystectomy as a comparator, papers that do not measure the learning curve from the outcomes, and case reports from the study types.

Information Sources and Search Strategy

The search included databases such as PubMed, Cochrane, and Embase. We used keywords and mesh terms to search the databases. The keywords and mesh terms are listed as cholecystectomy, gallbladder excision, gallbladder resection, robot surgery, robot-assisted, laparoscopic, keyhole, learning curve, proficiency curve, operating time, operative time, console time, and surgical time.

Automation tools excluding all papers before 1997 were used as that was the year the first robotic cholecystectomy was performed [6]. Two independent reviewers screened through the papers and agreed to the terms of the papers to include in concordance with the eligibility criteria.

Data was collected from March to April on a pre-made Excel spreadsheet, and data regarding the number of cases required for the learning curve to be achieved as well as the improvement in total operating time was sought. The range of total operating times as well as the values for the total operating time at the beginning and during the plateau was taken down. Other important information such as the experience of the surgeon, number of surgeons, total number of cases and study characteristics were also collected. A second reviewer double-checked the data collected.

ROBINS-I (Risk Of Bias In Non-randomized Studies - of Interventions) was used to assess the risk of bias in all studies. This was done by two reviewers who discussed any disagreements in results. ROBINS-I were used to display the results. A descriptive summary of the results was done, and no statistical analysis was done due to the heterogeneity of the papers.

Results

**Figure 1:**
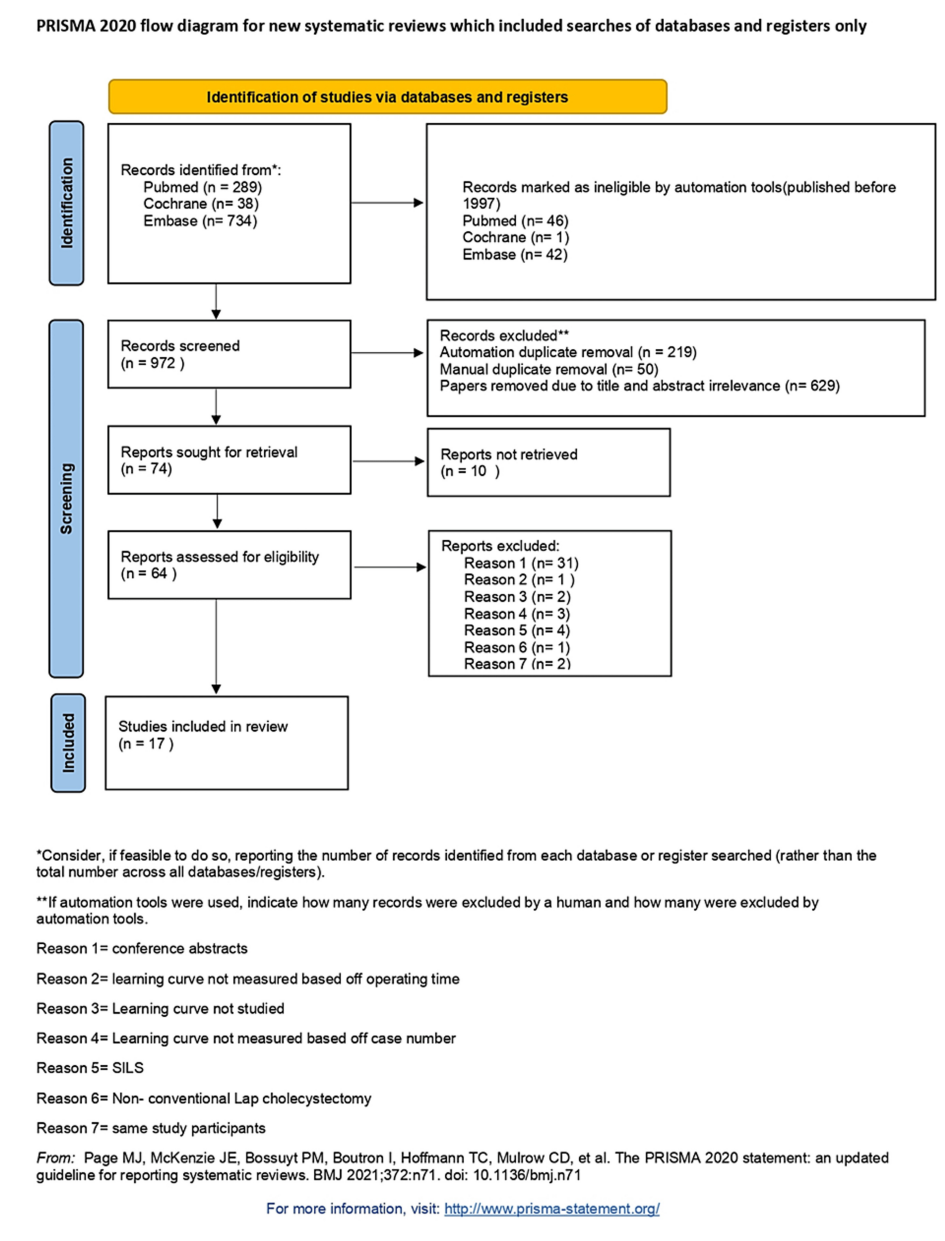
PRISMA flowchart PRISMA: Preferred Reporting Items for Systematic Reviews and Meta-Analyses; SILS: single incision laparoscopic surgery

**Figure 2:**
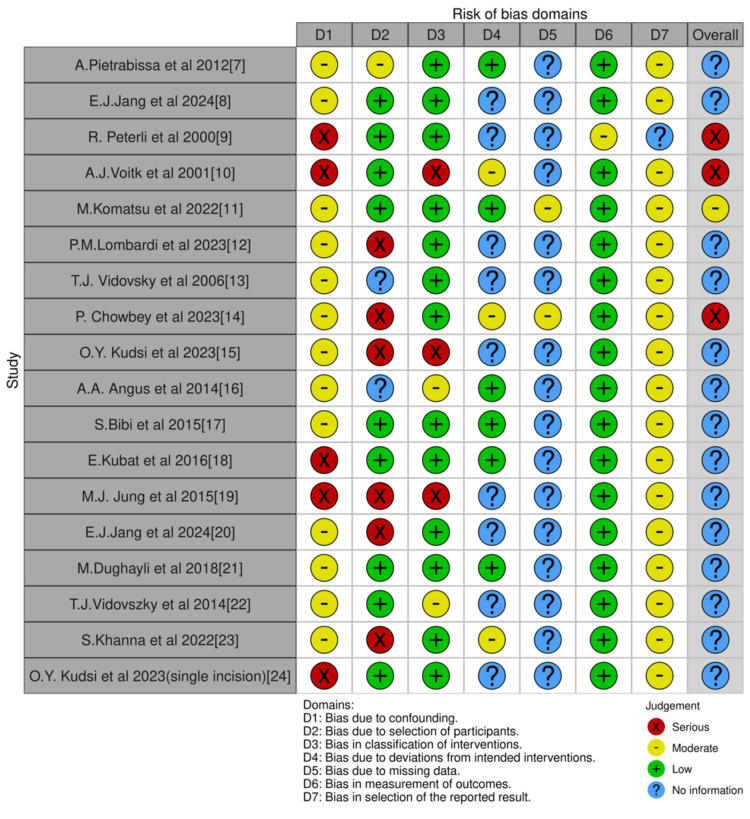
The ROBINS-I tool was used to assess the learning curve for robotic cholecystectomy versus laparoscopic cholecystectomy. ROBINS-I: Risk Of Bias In Non-randomized Studies - of Interventions

A total of 17 studies were included, all being non-RCTs. The majority were retrospective or prospective observational studies of single institutions. One study on SSRC was multi-institutional [7]. Four studies included CLC only, four studies included MRC only, eight studies included SSRC only and one study looked at SSRC but used CLC as a comparator for outcomes unrelated to the learning curve [8]. Figure 1 displays the final included studies based on PRISMA guidelines. Figure 2 shows the risk of bias by using ROBIN-I tools.

Learning Curve

Table 1 shows the study characteristics table for the learning curve of robotic and laparoscopic cholecystectomy, and Table 2 shows the learning curve of robotic cholecystectomy and laparoscopic cholecystectomy.

**Table 1:** Study characteristics comparing learning curves of robotic vs. laparoscopic cholecystectomy

No.	Authors	Year	Region	Study Design	Study Period	Group	Surgeons	Caseload
1	Pietrabissa et al. [7]	2012	Italy	Multi-institutional prospective longitudinal observational	1 March 2011-31 August 2011	Single site robotic	5	100
2	Jang et al. [8]	2023	Korea - Dong-A University	Retrospective observational single-centre	1 January 2019-31 December 2022	Single site robotic	1	117
3	Peterli et al. [9]	2000	Switzerland	Prospective series observational single-centre	July 1990-June 1997	Laparoscopic	7	2650
4	Voitk et al. [10]	2001	Canada	Prospective observational single-centre	1992-1998	Laparoscopic	1	500
5	Komatsu et al. [11]	2022	Japan	Retrospective single centre	January 2016-December 2020	Laparoscopic	16	514
6	Lombardi et al. [12]	2023	Italy (University of Milan)	Retrospective observational single-centre	2016-2021	Laparoscopic	6	340
7	Vidovszky et al. [13]	2014	USA (University of California)	Prospective cohort study observational single-centre	January 2012-January 2013	Single site robotic	N/A	95
8	Chowbey et al. [14]	2023	India	Observational single centre	October 2021-February 2022	Multiport robotic	1	100
9	Kudsi et al. [15]	2023	USA	Retrospective observational single-centre	2012-2021	Multiport robotic	1	245
10	Angus et al. [16]	2014	USA	Retrospective case series - observational single-centre	21 March 2012-24 August 2012	Single site robotic	1	55
11	Bibi et al. [17]	2015	USA	Retrospective evaluation of case series - observational single-centre	June 2012-January 2013	Single site robotic	1	102
12	Kubat et al. [18]	2016	USA	Retrospective review of prospective case series - observational single-centre	May 2012-August 2013	Single site robotic	1	150
13	Jung [19]	2015	Korea	Retrospective observational single-centre	October 2013-October 2014	Single site robotic	1	50
14	Jang and Kim [20]	2024	Korea - Dong-A University	Retrospective observational single-centre	April 2019-June 2023	Single site robotic	2	235
15	Dughayli et al. [21]	2018	USA - Henry Ford Wyandotte Hospital	Retrospective observational single-centre	March 2013-April 2015	Single site robotic	2	117
16	Vidovsky et al. [22]	2006	USA (University of California)	Prospective case series	July 2004-December 2005	Multiport robotic	N/A	51
17	Khanna and Barua [23]	2022	India	Retrospective cohort single-centre	2 February 2020-18 August 2021	Multiport robotic	1	106
18	Kudsi et al. [24]	2023	USA	Retrospective observational single-centre	February 2012-July 2021	Single site robotic	1	259

**Table 2:** Learning curve of robotic cholecystectomy and laparoscopic cholecystectomy CLC: conventional laparoscopic cholecystectomy

Study	Year	Group	Surgeon(s) Prior Experience	Tail of the Learning Curve Based on Operating Time (Cases)
Pietrabissa et al. [7]	2012	Single site robotic	Each general surgeon had >100 cases each, each team had one day of training with animals in the laboratory	No learning curve exhibited - flat line
Jang et al. [8]	2023	Single site robotic	First experience with single-port robotic	18-53
Peterli et al. [9]	2000	Conventional laparoscopic	Residents and staff surgeons	15-20
Voitk et al. [10]	2001	Conventional laparoscopic	General surgeon in the sixth decade	200
Komatsu et al. [11]	2022	Conventional laparoscopic	Sixteen surgeons, 10 surgeons first time performing laparoscopic cholecystectomy. Four had performed laparoscopic cholecystectomy before	60
Lombardi et al. [12]	2023	Conventional laparoscopic	Newbies with no laparoscopic cholecystectomy experience	7-25
Vidovszky et al. [13]	2006	Multiport robotic	Attending, chief resident, minimally invasive surgical fellow	16-32
Chowbey et al. [14]	2023	Multiport robotic	N/A	30
Kudsi et al. [15]	2023	Multiport robotic	Trained in minimally invasive	84-134
Angus et al. [16]	2014	Single site robotic	Robotic novice with 4000-5000 cases of laparoscopic cholecystectomy	40
Bibi et al. [17]	2015	Single site robotic	Extensive experience in laparoscopic cholecystectomy	20
Kubat et al. [18]	2016	Single site robotic	Experienced minimally invasive surgeon	48
Jung et al. [19]	2015	Single site robotic	N/A	40 (actual dissection time 20)
Jang et al. [20]	2024	Single site robotic	Expert surgeon with 2000 CLC cases, novice surgeon with 100 CLC cases	Expert: 15 Novice:10
Dughayli et al. [21]	2018	Single site robotic	Surgeon 1 had experience in advanced laparoscopic skills and more than 10 years of experience, surgeon 2 had basic laparoscopic skills	>102
Vidovsky et al. [22]	2014	Single site robotic	N/A	No learning curve exhibited
Khanna et al. [23]	2022	Multiport robotic	40 years of experience in biliary surgery	No learning curve exhibited
Kudsi et al. [24]	2023	Single site robotic	The surgeon has performed 160 multiport robotic cholecystectomies	91

This was reported in 14 studies with three on MRC, seven on SSRC and four on CLC. The rest of the studies did not exhibit a learning curve and had non-significant changes in their operating time throughout the study.

For SSRC, the number of cases required for the learning curve to be achieved ranged from 200 to as little as 7 [9-12]. Out of the four studies on MRC, only three of them reported the total operating time to plateau after a certain number of cases [9-11,13-15]. The number of cases required for the operating time to plateau ranges from 134 to 16 over the three studies. One study involving a surgeon with 40 years of biliary surgery experience did not exhibit a learning curve stating it was non-existent in the study.

Out of the nine studies on SSRC, seven reported the number of cases required for the learning curve to be achieved based on total operating time [8,16-19]. The number of cases ranged from as little as 10 and going beyond 102 cases [21]. Ten cases were reported in the study comparing a novice surgeon and an expert surgeon, this number was achieved by the novice. Two studies did not report a learning curve, one was a multi-institutional study looking at five different surgeons from separate centres with >102 cases each of da Vinci surgery. It displayed a flat line in terms of operating time in SSRC [7]. All four studies on CLC reported on the number of cases required for the operative time to plateau. Another study revealed that the total operative time remained unchanged throughout the study with only some statistical significance in changes with setup time or robotic time in obese patients [22].

Improvement in Operating Time

Table 3 shows the improvement in operating time for robotic cholecystectomy and laparoscopic cholecystectomy.

**Table 3:** Operating time for robotic and laparoscopic cholecystectomy

Study	Year	Group	Operating Time Range	Improvement in Operating Time
Pietrabissa et al. [7]	2012	Single port robotic	Total operative time range 140-39 mins; Console cholecystectomy time range 80-12 mins	N/A
Jang et al. [8]	2023	Single port robotic	N/A	N/A
Peterli et al. [9]	2000	Conventional laparoscopic	N/A	Started at 100 mins operating time, decreased and remained at around 71 minutes
Voitk et al. [10]	2001	Conventional laparoscopic	Total surgical time range: 117 mins-14 mins	Mean operative time for the first 20 cases is 52 mins. First 100 cases: 44 mins, last 100 cases: 32 mins
Komatsu et al. [11]	2022	Conventional laparoscopic	Total surgical time range: 279-28 mins	Median of 112 mins for first case, median of 64 mins after 60 cases
Lombardi et al. [12]	2023	Conventional laparoscopic	N/A	N/A
Vidovsky et al. [13]	2006	Multiport robotic	N/A	Average of 85.6 mins in the initial phase, an average of 68.2 mins in the advanced phase, significant improvement between stages p=0.0055
Chowbey et al. [14]	2023	Multiport robotic	Console time range: 87-10 mins	Mean surgical time for first 50 cases: 28.53 mins, last 50 cases: 22.06 mins
Kudsi et al. [15]	2023	Multiport robotic	N/A	Average skin-to-skin time of early group: 61.17 mins, middle phase: 46.67 mins, late phase: 44.41 mins. P=0.0001
Angus et al. [16]	2014	Single port robotic	Average total OR time range 105-40 mins; mean console time range 66-15 mins	N/A
Bibi et al. [17]	2015	Single site robotic	Total operative time range 265-36 mins; console time range 179-26 mins	Mean total operative time first 20 cases was 141 mins, last 20 cases 85 mins, P=0.001. Mean total console time first 20 cases 102 mins, last 20 cases 40 mins, P=0.001
Kubat et al. [18]	2016	Single port robotic	Total operative time range 212-32 mins; elective total operative time range 212-32 mins; urgent total operative time range 119-32 mins	N/A
Jung et al. [19]	2015	Single port robotic	N/A	N/A
Jang et al. [20]	2024	Single port robotic	N/A	N/A
Dughayli et al. [21]	2018	Single port robotic	N/A	N/A
Vidovsky et al. [22]	2014	Single port robotic	N/A	Total operating time first 16 cases 85.6 ± 12.7 mins, last 16 cases 68.2 ± 17.1 min. Robotic operative time first 16 cases 38.2 ± 22.9 min, last 16 cases 32.5 ± 12.7 min
Khanna et al. [23]	2022	Multiport robotic	Console time range 205-6 mins	Mean console time for first 50 cases: 51.1 mins, next 56 cases: 54.16 mins/mean set up time for first 50 cases: 10.66 mins, next 56 cases: 8.07 mins
Kudsi et al. [24]	2023	Single port robotic	Skin-to-skin time range 62.5-25.5 mins; robotic console time range 36-12 mins	Mean skin-to-skin time in the early phase first 91 cases 53.8 mins, late phase last 168 cases 30.0 mins, P<0.0001 Mean robotic console time in the early phase 24 mins, late phase 15 mins, P<0.001

Only two of the MRC studies recorded the ranges of operating times [14,23]. Both studies only recorded the range for the robotic console and set up time individually. Overall the range for robotic console time was 205-6 minutes. All four MRC studies reported on the improvement in times taken with one study stating an average of 85.6 minutes in the first 16 cases and 68.2 minutes in the last 15 cases [14]. Two studies recorded the average of times for the first half (50 cases) and the second half of cases. A study had an average robotic console time of 51.1 minutes which increased to 54.16 minutes for the last 56 cases. This study by Khanna and Barua did not exhibit a learning curve [24]. The other study had an average of 28.53 minutes for the first 50 cases and improved to 22.06 minutes in the next 50 cases [14]. The last study on MRC showed an improvement from an average of 61.17 minutes in the early phase (1-84 cases), 46.67 minutes in the middle phase (85-134 cases) to 44.41 minutes in the late phase (135-245 cases) [15].

Five SSRC studies reported on the ranges of total operating times. Overall it varied from 265 to 25.5 minutes [7,16-18,24]. The range for SSRC console times was only reported by four of the studies which ranged from 179 to 12 minutes. Improvement in times was reported in three of the SSRC studies, a study looking at the first 16 and last 16 cases showed an improvement from 85.6 to 68.2 minutes for total operating time. Console times showed improvement from 38.2 to 32.5 minutes [22]. One study looking at the first 20 cases and the last 20 cases showed improvement from 141 to 85 minutes for total operative time [17]. Console time improved from 102 to 40 minutes. The last SSRC that measured change in operating time looked at the first 91 cases and the last 168 cases. An improvement of 53.8 minutes to 30 minutes was shown. Console time improved from 24 to 15 minutes [24].

Two of the studies on CLC reported the range of operating times in their studies [10,11]. Time taken ranged from 249 minutes to 14 minutes. Three studies reported an improvement in operating time [9-12]. One study had the study starting off with 100 minutes and later improved to 71 minutes where the improvement plateaued [9]. Another study reported 52 minutes as the mean operative time for the first 20 cases, which improved to 44 minutes for the first 100 and finally 32 minutes for the last 100 [10]. The last study on CLC reporting on improvement in total operating time recorded the median of surgeons' first time operating which was 112 minutes. This improved to a median of 64 minutes after 60 cases where the improvement started to plateau [11].

Discussions

Studies from both the multiport robotic and single incision robotic cholecystectomy have shown a varied number of cases required. This may be due to the high levels of heterogeneity between the studies with surgeons of different experiences. The study by Pietrabissa et al. (2012) described the learning curve as already achieved prior to the study resulting in the flat line with increasing experience of cases [7] suggesting that the learning curve was very short. However, this could possibly be due to their prior experience in MRC rather than the one day of lab training completing their learning curve. Although experience in MRC did not always guarantee a shorter learning curve for SSRC, one of the studies involving a robotic surgery novice had a learning curve of 40 [16] whilst a surgeon with 160 cases experience of in MRC required 91 cases.

In comparison, CLC has given the largest number of cases required for the operating times to level off as shown in Voitk et al. (2001) reporting 200 cases being required [10]. However, it has also been shown to have required the least number of cases as shown in the study by Lombardi et al. (2023) where one of the surgeons in the study was able to achieve proficiency in as little as seven cases [12]. This could possibly be related to CLC being a well-established technique in 2023 compared to in 2001 where CLC was relatively still novel. Although the study from 2023 was done by newbies, no information was given regarding whether they had prior experience with simulator training or laparoscopic box trainers. These have become increasingly popular over the years and may not have been as accessible in 2001. There is no clear trend in the number of cases for proficiency in CLC.

SSRC had the greatest range in total operating times with MRC having the smallest range. The improvements in operating times were all reported in different ways. Averages were taken over different numbers of cases for both the start and the plateau of the learning curve making it difficult for comparisons to be made regarding the improvement in time taken.

This study has several limitations mainly due to the scarcity of studies. All of the studies are non-RCTs that only follow the learning curve of a singular surgeon. The small sample size of surgeons allows for results to be easily skewed by anomalies. None of the studies were RCTs and none provided a direct comparison between CLC and robotic cholecystectomy in terms of the learning curve. The risk of bias showed many of the studies used had serious levels of bias. In addition to this, 10 studies had to be discarded due to not having access, thus removing possibly eligible studies.

## Conclusions

Methods such as CLC, MRC and SSRC have shown variable values for the learning curve. However, the lack of homogeneity and high-quality studies makes such results inconclusive. Studies with a greater sample size of surgeons and better control over the surgeon experience would benefit future reviews. Considering MRC and SSRC are both still fairly novel, there is an opportunity for more RCTs and higher quality studies to be made which will hopefully provide conclusive evidence regarding the learning curve of robotic cholecystectomy in comparison to CLC.
